# Single center experience on talc poudrage morbidity: focus on high talc dosage

**DOI:** 10.1186/1749-8090-6-87

**Published:** 2011-06-27

**Authors:** Giovanni Leuzzi, Maria Letizia Vita, Venanzio Porziella, Maria Teresa Congedo, Alfredo Cesario

**Affiliations:** 1 Department of Thoracic Surgery, Catholic University of Sacred Heart, Rome, Italy; 2 CdC San Raffaele Velletri, Rome, Italy

## Abstract

Malignant pleural effusion (MPE) is a common clinical problem of concern for most of the pneumologists and thoracic surgeons. A general consensus regarding the use of talc poudrage in treatment of MPE exists, but only few studies analyzed in detail talc insufflation related pulmonary morbidity. In particular, ARDS talc-related is caused by physical and chemical effects of the small talc particles (50% particle size <15 μm) and its occurrence is independent from the underlying disease, the quantity of talc used or the technique of talc instillation. In our series we observed 3 cases only (0.75%) of talc-related lung injury. This data strongly confirm the low rate of talc-related lung injury after talc poudrage in treatment of MPE regardless the amount of talc insufflated.

## Letter to the editor

We read with great interest the report by Barbetakis et al [1] on morbidity, mortality and life expectancy following thoracoscopic talc insufflation. Malignant pleural effusion (MPE) is a common clinical problem of concern for most of the pneumologists and thoracic surgeons. In the US the annual incidence of MPE is estimated to be 250.000 cases and several studies suggest that exudative effusions are by large (42 - 77%) caused by malignancy [2]. A general consensus regarding the use of talc poudrage in treatment of MPE exists, only few studies analyzed in detail talc insufflation related pulmonary morbidity. The most common side effects are pleuritic chest pain and mild fever. However very serious and potentially fatal adverse events, albeit unusual (1 to 9% in published series [3]), as such as an adult respiratory distress syndrome (ARDS), can happen and these are related to the physical and chemical effects of the small talc particles (50% particle size <15 μm).

The occurrence of ARDS is, in fact, independent from the underlying disease, the quantity of talc used or the technique of talc instillation but it's strongly related to talc particle size. A greater alveolarearterial oxygen gradient in the group exposed to non-graded talc at 48 h after pleurodesis was detected when matched with that of those exposed to graded talc [4].

In our department, in the period between 01/95 and 10/10, we performed thoracoscopic talc pleurodesis in 401 patients with MPE. As previously reported in our data [5], we performed a "single access" technique in those cases with no evidence of pleural adhesions or loculated effusions (241 patients), while 160 patients underwent a standard thoracoscopy. We routinely used an average of 4 grams (range 2-28 grams) of large-particle asbestos-free talc pneumatically atomized through a soft silicone tube. To be specified that the upper range (up to 28 grams) was reached in those cases (15 patients) with high (up to 500 ml/die) flow. In our series we observed 3 cases only (0.75%) of talc-related lung injury (acute respiratory failure in 2 cases and acute pulmonary edema in 1 patient). Our complication rate is lower to that reported in [1] (acute respiratory failure in 7 cases and reexpansion pulmonary edema in 1 patient), although the Authors have denied a correlation between talc insufflation and pulmonary complications. As well, differently from the evidence reported by Montes et al [6], we did not observe any complications in the 15 cases insufflated with more than 8 grams of talc.

Our results confirm the low rate of talc-related lung injury after talc poudrage in treatment of MPE regardless the amount of talc insufflated. We would really appreciate the Authors' reflection and reaction in considering and discussing about high-dose (over 8 g) graded-talc pleurodesis.

## Competing interests

The authors declare that they have no competing interests.

## Authors' contributions

GL conceived the study, collected data and drafted the manuscript. MLV reviewed the pertinent literature. VP and MTC helped with bibliography. AC critically revised the paper. All authors read and approved the final version of the manuscript

## References

[B1] Barbetakis N Asteriou C Papadopoulou F Samanidis G Paliouras D Kleontas A Lyriti K Katsikas I Tsilikas C Early and late morbidity and mortality and life expectancy following thoracoscopic talc insufflation for control of malignant pleural effusions: a review of 400 cases J Cardiothorac Surg 2010 5 27 10.1186/1749-8090-5-27 20403196 PMC2873359

[B2] Anon Management of malignant pleural effusions Am J Respir Crit Care Med 2000 162 1987e2001 10.1164/ajrccm.162.5.ats8-00 11069845

[B3] Sahn SA Light RW Pro/con editorial: talc should/should not be used for pleurodesis Am J Respir Crit Care Med 2000 162 2023 6 11112103 10.1164/ajrccm.162.6.pc09-00a

[B4] Maskell NA Lee YC Gleeson FV Randomized trials describing lung inflammation after pleurodesis with talc of varying particle size Am J Respir Crit Care Med 2004 170 377e82 10.1164/rccm.200311-1579OC 15142871

[B5] Margaritora S Cesario A Vita ML Granone P Single versus multiple access video-assisted thoracic surgery in the treatment of malignant pleural effusion Eur J Cardiothorac Surg 2007 32 2 397 author reply 397-8. Epub 2007 Jun 13 17566752 10.1016/j.ejcts.2007.05.007

[B6] Montes JF Ferrer J Villarino MA Influence of Talc Dose on Extrapleural Talc Dissemination after Talc Pleurodesis Am J Respir Crit Care Med 2003 168 348 55 10.1164/rccm.200207-767OC 12773332

